# Carriage and Gene Content Variability of the pESI-Like Plasmid Associated with *Salmonella* Infantis Recently Established in United States Poultry Production

**DOI:** 10.3390/genes11121516

**Published:** 2020-12-18

**Authors:** Elizabeth A. McMillan, Jamie L. Wasilenko, Kaitlin A. Tagg, Jessica C. Chen, Mustafa Simmons, Sushim K. Gupta, Glenn E. Tillman, Jason Folster, Charlene R. Jackson, Jonathan G. Frye

**Affiliations:** 1 Bacterial Epidemiology and Antimicrobial Resistance Research Unit, U.S. Department of Agriculture, Agricultural Research Service, U.S. National Poultry Research Center, Athens, GA 30605, USA; Elizabeth.Mcmillan@usda.gov (E.A.M.); Charlene.Jackson@usda.gov (C.R.J.); 2 Eastern Laboratory, U.S. Department of Agriculture, Food Safety and Inspection Service, Athens, GA 30605, USA; jamie.wasilenko@usda.gov (J.L.W.); mustafa.simmons@usda.gov (M.S.); glenn.tillman@usda.gov (G.E.T.); 3 Enteric Diseases Laboratory Branch, Centers for Disease Control and Prevention, Atlanta, GA 30333, USA; nnp2@cdc.gov (K.A.T.); lly3@cdc.gov (J.C.C.); gux8@cdc.gov (J.F.); 4 Weems Design Studio, Inc., Suwanee, GA 30024, USA; 5 Department of Biochemistry and Molecular Biology, Oklahoma State University, Stillwater, OK 74078, USA; sushim.gupta@okstate.edu

**Keywords:** *Salmonella*, infantis, plasmid, antibiotic resistance

## Abstract

*Salmonella* Infantis carrying extended spectrum β-lactamase *bla*_CTX-M-65_ on a pESI-like megaplasmid has recently emerged in United States poultry. In order to determine the carriage rate and gene content variability of this plasmid in U.S. *Salmonella* Infantis, whole genome sequences of *Salmonella* isolates from humans and animals in the U.S. and internationally containing the pESI-like plasmid were analyzed. The U.S. Department of Agriculture Food Safety and Inspection Service (FSIS) identified 654 product sampling isolates containing pESI-like plasmids through hazard analysis and critical control point (HACCP) verification testing in 2017 and 2018. The Centers for Disease Control and Prevention identified 55 isolates with pESI-like plasmids in 2016–2018 through the National Antimicrobial Resistance Monitoring System. Approximately 49% of pESI-like plasmids from FSIS verification isolates and 71% from CDC NARMS contained *bla*_CTX-M-65_. Pan-plasmid genome analysis was also performed. All plasmids contained *traN* and more than 95% contained 172 other conserved genes; 61% contained *bla*_CTX-M-65_. In a hierarchical clustering analysis, some plasmids from U.S. animal sources clustered together and some plasmids from South America clustered together, possibly indicating multiple plasmid lineages. However, most plasmids contained similar genes regardless of origin. Carriage of the pESI-like plasmid in U.S. appears to be limited to *Salmonella* Infantis and carriage rates increased from 2017 to 2018.

## 1. Introduction

*Salmonella enterica* is one of the leading causes of foodborne illness in the United States and around the world [1,2]. While there are over 2500 serotypes of *Salmonella*, 20 serotypes, including serotype Infantis, account for most U.S. human infections [3]. Infantis was the sixth most common serotype associated with human infections in 2016 and the fifth most common serotype isolated from chicken products in the U.S. in 2014 [3,4]. Infantis has consistently been in the top ten serotypes isolated from chicken since 1998 [5]. Incidence of laboratory-confirmed human salmonellosis cases as a result of *S*. Infantis have increased in recent years as well [3,6]. An outbreak of *S.* Infantis carrying the extended spectrum β-lactamase (ESBL) gene *bla*_CTX-M-65_ associated with raw chicken in the U.S. was reported in January of 2018. Infections were initially source-tracked with pulsed-field gel electrophoresis (PFGE) pattern JFXX01.0787. In total, 129 people from 32 states were infected and one death was reported. While the investigation concluded in January of 2019, the outbreak was never linked to one specific chicken source [7].

ESBL-producing Enterobacteriaceae are considered a serious threat to human health [8]. ESBL genes, which confer resistance to third generation cephalosporins as well as other cephalosporins, were considered rare in *Salmonella* isolated from humans and animals in the U.S. until recently [9]. Although ESBL genes are identified more frequently in human clinical isolates in the U.S., ESBL-producing strains have emerged in U.S. food animal populations, with *S*. Infantis strains most prominent [10]. Third generation cephalosporins are used for treatment of severe salmonellosis cases, thus strains exhibiting resistance to these drugs have limited treatment options [11].

*S.* Infantis strains containing *bla*_CTX-M-65_ have been detected world-wide [12,13,14,15,16]. International and domestic isolates are associated with a conjugative megaplasmid (~300 kb) containing sequence associated with three incompatibility groups, similar to the pESI and pESI-like plasmids first described in *S*. Infantis strains in Israel. This plasmid also contains several factors that could confer a survival advantage compared to other *Salmonella* strains, such as the yersiniabactin iron acquisition operon and several fimbriae that allow for increased attachment to human and poultry epithelial cells [17,18].

Researchers determined that a variant of the pESI plasmid was circulating in domestic *S.* Infantis strains as early as 2012. This plasmid was shown to carry the *bla*_CTX-M-65_ gene as well as several other antimicrobial resistance genes [19,20]. However, the carriage rate of this plasmid in the U.S. is unknown. In order to gain a better understanding of the pESI-like plasmid in the U.S., we sought to determine the carriage rate and variation in gene content of the pESI-like plasmid in *Salmonella* from humans and food animal products in the U.S. and international sources.

## 2. Materials and Methods

### 2.1. Plasmid Carriage

Whole genome sequence (WGS) data of non-typhoidal *S. enterica* isolates collected from product samples by the U.S. Department of Agriculture Food Safety and Inspection Service (USDA-FSIS) at federally inspected slaughter and processing facilities for the hazard analysis and critical control point (HACCP) program were analyzed for the presence of the pESI plasmid. Isolates to be included in the 2017 and 2018 National Antimicrobial Resistance Monitoring System (NARMS) integrated report were investigated; isolates from 2015 and 2016 were not analyzed because WGS data was not available for all isolates at that time. Isolation procedures are described in the USDA-FSIS Microbiology Laboratory Guidebook (MLG) Chapter 4 [21].

WGS surveillance data from human clinical cases of *S. enterica*, *Escherichia coli*, and *Shigella* sp. isolates collected by the Centers for Disease Control and Prevention (CDC) for the NARMS program in 2016–2018 were analyzed for the presence of the pESI plasmid. WGS data of *S. enterica* from 2015 was also analyzed. Sampling procedures are described in the 2015 NARMS Report [22]. As CDC datasets are not yet finalized, total numbers of isolates may differ from future NARMS reports. In order to confirm the serotype provided by the submitting public health laboratory of one CDC *Salmonella* isolate, WGS data was analyzed using SeqSero 2, accessed through the Center for Genetic Epidemiology and used to determine the serotype (http://www.genomicepidemiology.org/) [23].

WGS assemblies were screened for 14 target sequences indicative of presence of the pESI plasmid using the basic local alignment search tool (BLAST) (Table 1, Supplementary Table S1) [24]. Target sequences were adapted from PCR targets designed in a 2015 study of Italian *S*. Infantis isolates that contained the pESI plasmid. Targets sequences included target genes for the IncI1 pMLST scheme, a hypothetical protein, fimbriae, and a gene from the yersiniabactin (*Ybt*) operon that were shown to be associated with the plasmid in Italian isolates [12]. Additional target sequences for the *bla*_CTX-M-65_ gene, pESI specific *repA*, I1 relaxase, and the IncP replicon-associated sequences were designed and added in this manuscript as they were determined to also indicate pESI plasmid presence (Table 1, Supplementary Table S1). Isolates with greater than 95% coverage and identity to the target sequence were considered to contain the target. Isolates were considered to contain the pESI plasmid only if they contained the pESI specific *rep*A gene plus five additional target sequences. Plasmids were not classified as partial or complete. Carriage rate was calculated using the formula below, and only for isolates of serotype Infantis for each source type.

$$\mathrm{Carraige}\;\mathrm{Rate}\;=\;\frac{\mathrm{Isolates}\;\mathrm{containing}\;\mathrm{the}\;\mathrm{pESI}\;repA\;\mathrm{gene}\;\mathrm{and}\;\mathrm{five}\;\mathrm{additional}\;\mathrm{targets}}{\mathrm{Total}\;\mathrm{Isolates}\;}$$

### 2.2. Gene Content Analysis

In order to explore the diversity of genes associated with the pESI plasmid in the U.S. with the fewest limitations, all *S*. Infantis sequences from FSIS product sampling found in the Enterobase database (https://enterobase.warwick.ac.uk/) associated with BioProject number PRJNA242847, the number for GenomeTrakr, prior to 30 June 2019 were investigated [25]. Only isolates uploaded by FSIS associated with the BioProject were included. For isolates containing the pESI plasmid, sequences were extracted to create a bin of contigs belonging to the plasmid (positive, *n* = 1323). For comparison, isolates sharing the same NCBI SNP cluster as the FSIS product sample isolates, defined by NCBI’s Pathogen Browser, as of 23 September 2019, were also analyzed in the same manner. These isolates included those sequenced as part of the CDC-PulseNet program prior to 30 June 2019 (*n* = 801), and isolates collected in other countries (*n* = 503). Countries of origin included Chile (*n* = 29), Colombia (*n* = 53), Ecuador (*n* = 206), Peru (*n* = 157), Switzerland (*n* = 3), United Kingdom (*n* = 52), Venezuela (*n* = 2), and Vietnam (*n* = 1).

Closed pESI-like plasmids sequenced by FDA (*n* = 4) were used to create a custom BLAST database for identifying and binning plasmid sequences from isolates downloaded from Enterobase and NCBI [19]. Contigs with greater than 98% identity and more than 70% coverage to these pESI sequences were considered belonging to the pESI plasmid and extracted from the WGS and considered part of the pESI-like plasmid bin. Coverage did not have to be continuous, as larger contigs were often split into two “hits” that allowed for coverage equal to or greater than 70%. Contigs of less than 300 bp that did not have continuous coverage were excluded due to possible assembly error.

Plasmid bins were annotated based on the closed pESI-like sequences using Prokka v1.12 [26]. Annotated plasmid bins were analyzed using Roary version 3.11.2 (https://sanger-pathogens.github.io/Roary/) to determine the genes common to all plasmids [27]. Roary was run without paralog splitting to prevent creation of erroneous gene clusters. Genes with 95% sequence identity were grouped together. Gene clusters identified by Roary were annotated using BLAST against the non-redundant protein database, curated by NCBI.

### 2.3. Hierarchal Clustering and Dendrogram Visualization

Using the presence/absence table generated by Roary, plasmid bins were clustered based on the presence and absence of genes that were found in two or more isolates (*n* = 649 genes). Hierarchical clustering (single linkage) based on the Jaccard Index was calculated using the vegan package in R and visualized with FigTree (https://github.com/rambaut/figtree/releases/tag/v1.4.4) [28].

## 3. Results

### 3.1. Plasmid Carriage

**Figure 1:**
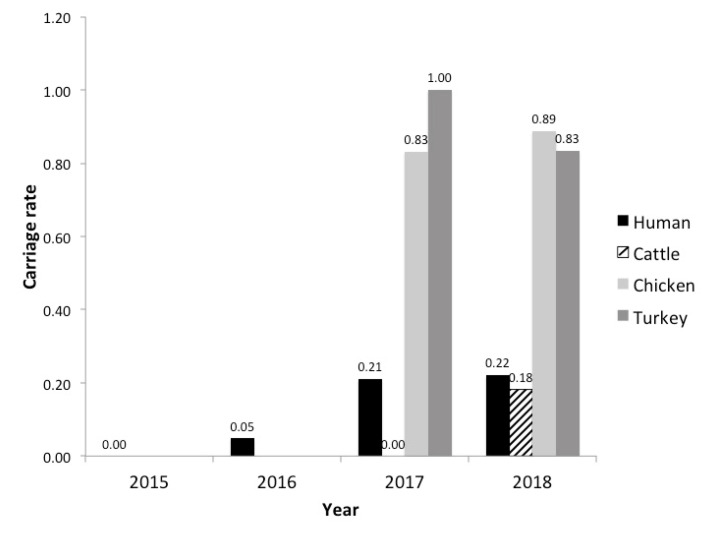
Carriage rate of the pESI plasmid in *Salmonella* Infantis chicken, turkey, and cattle isolates from USDA-FSIS and human isolates from CDC NARMS. 2015 and 2016 data was not analyzed for USDA-FSIS isolates. Total number of Infantis isolates for each year are as follows: *n* = 73 humans 2015, *n* = 83 humans 2016, *n* = 100 humans 2017, *n* = 113 humans 2018; *n* = 5 cattle 2017, *n* = 11 cattle 2018; *n* = 314 chickens 2017, *n* = 389 chickens 2018; *n* = 9 turkeys 2017, *n* = 18 turkeys 2018.

Non-typhoidal *Salmonella* sequences (*n* = 4961) collected by USDA-FSIS included in the NARMS datasets in 2017 (*n* = 2442) and 2018 (*n* = 2519) from three commodities, cattle (*n* = 532), chicken (*n* = 4016), and turkey (*n* = 413), were screened for target sequences of the pESI-like plasmid. In total, 654 isolates contained a pESI-like plasmid, 606 of which were Infantis isolated from chickens; 22 were a rough variant of Infantis (Rough O:r:1,5) from chickens [29]. Twenty-four Infantis isolates from turkey contained the pESI plasmid and two Infantis from cattle. By animal host source, 86.2% (*n* = 606/703) of Infantis isolates from chickens and 88.8% (*n* = 24/27) of Infantis isolates from turkeys contained the plasmid. In contrast, only 12.5% (*n* = 2/16) of Infantis isolates from cattle contained the plasmid. The carriage rate of the plasmid increased from 2017 to 2018 in chickens and cattle but decreased in turkey (Figure 1).

Only 49% (*n* = 320/654) of isolates with the plasmid contained *bla*_CTX-M-65_. No isolates with a plasmid contained the hypothetical backbone protein target (Table 2). In isolates without the *ipf* and K88 targets, a partial copy of each target was detected but the coverage was too low to be considered containing the target sequence; coverage ranged between 88 and 94% for the *ipf* target and between 68 and 94% coverage for the K88 target.

A total of 11,311 *Salmonella* isolates from humans submitted to CDC in 2015 (*n* = 2377), 2016 (*n* = 2857), 2017 (*n* = 2937), and 2018 (*n* = 3140) for the NARMS program were screened for the pESI plasmid. These included both typhoidal and non-typhoidal isolates. Of those, 369 were serotype Infantis; 73 from 2015, 83 from 2016, 100 from 2017, and 113 from 2018. No isolates from 2015 contained a pESI-like plasmid. Four isolates from 2016 contained a pESI plasmid, three contained all of the targets tested except for *bla*_CTX-M-65_, and one was also missing the hypothetical backbone protein target; all were serotype Infantis (*n* = 4/83 Infantis isolates; Table 3). One *S*. Berkeley isolate from 2016 contained the pESI *repA* gene and four additional target sequences and thus was not considered to contain the plasmid.

In 2017, 21 isolates from humans contained pESI-like plasmids (*n* = 21/100 Infantis isolates). Fourteen of the 21 contained all the targets except the hypothetical backbone protein. One isolate was reported as serotype Nigeria and to confirm the result, WGS data and SeqSero were used. The isolate was predicted to be an Infantis variant rather than serotype Nigeria. In 2018, 30 isolates contained pESI-like plasmids, 25 were serotype Infantis. Four isolates were serotype I 6,7,:-:,1,5 and one was Rough 1, 5; both serotypes can be variants of serotype Infantis (Table 3). Carriage rate of the plasmid increased in these human infection isolates from 2016 to 2018 and 71% contained *bla*_CTX-M-65_ (Figure 1, Table 3). No *E. coli* or *Shigella* isolates contained complete or partial pESI plasmids although some isolates did contain some of the target sequences.

### 3.2. Gene Content Analysis

Plasmid bins from all sources were analyzed using Roary to determine the most common genes carried by the pESI-like plasmid. A total of 2627 plasmids were included in the analysis (Supplementary Table S2). Only one gene, *traN*, normally associated with IncI1 plasmids, was detected in every isolate; 649 total genes were detected in at least two plasmids. Genes only detected in one isolate were ignored because of the possibility of assembly or annotation error influencing the gene’s inclusion.

**Figure 2:**
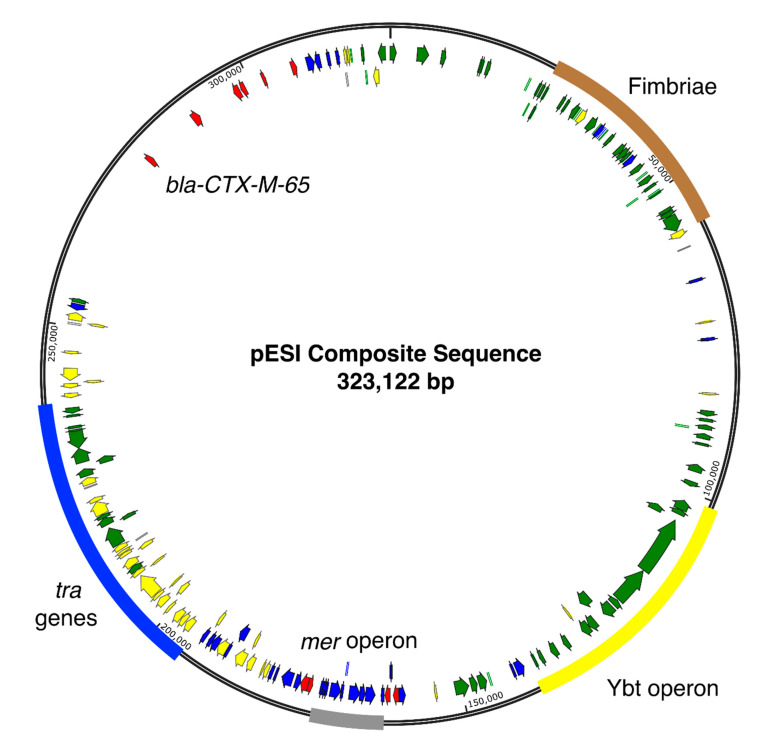
Composite sequence of the pESI plasmid. Genes detected in more than 90% of isolates arranged according to CP016407.1. Each arrow represents a gene present in at least 90% of isolates or an antibiotic resistance gene. Green arrows indicate genes detected in more than 99% of isolates. Yellow arrows represent genes detected in greater that 95% of isolates. Blue arrows represent genes detected in more than 90% of isolates. Antibiotic resistance genes are represented by red arrows, regardless of detection frequency. Regions of important genes are labeled.

In addition to *traN*, 100 other genes were detected in greater than 2601 (99%) plasmids including six of 12 target genes used to identify the pESI plasmid in the carriage rate analysis: pESI specific *repA*, *trbA*, I1 relaxase, *ipf*, a portion of the K88 target, and the Ybt target *irp*2. Other genes detected included conjugation genes, plasmid maintenance genes, fimbriae, toxin-antitoxin genes, transposases and hypothetical genes (Supplementary Table S3, Figure 2).

An additional 71 genes were detected in greater than 95% of plasmids. The *pilL*, *sogS*, and *ardA* targets were detected in this group. Other genes included conjugation genes, pili genes, transposases, and hypothetical proteins (Supplementary Table S4, Figure 2). The *merA* gene was detected in more than 90% of plasmids as well as *merD*, *merE*, *merP*, *merT*, and *merR*; *merC* was detected in slightly less than 90% of plasmids (Supplementary Table S5, Figure 2). Approximately 80% of plasmids contained arsenic resistance genes *arsH*, *arsA*, and *arsD*. The IncP target sequence was split and called two separate genes, both of which were detected in less than 35% of plasmids. The hypothetical backbone target was only detected in 3% of plasmids, which were all collected in Peru prior to 2013.

The *tetR* and *tetA* genes were detected in the most plasmids as compared to other antibiotic resistance (AR) genes (92.7%, *n* = 2436 and 2435 respectively). The *sul1* gene was the next most common AR gene (92%, *n* = 2423). Approximately 61% of the plasmids contained *bla*_CTX-M-65_ (*n* = 1602). Eight plasmids, all in isolates collected from Peru, contained an ESBL gene more similar to *bla*_CTX-M-186_ than *bla*_CTX-M-65_. The two variants are 79% similar at the amino acid level. Six other resistance genes, *aac3-VI*, *aph*4-Ia, *aadA*, *floR*, *dfrA14*, and *aph(3′)Ia*, were detected in 55–72% of plasmids (Supplementary Table S5, Figure 2). An additional 565 plasmids contained another variant of *aadA* and 104 plasmids contained both variants; at least one variant was detected in 92% of plasmids.

Figure 2 is the composite sequence of the pESI plasmid, showing the locations of genes detected in greater than 90% of plasmids relative to the location of the genes in CP016407.1 (pESI plasmid collected in 2015 from a chicken isolate). Locations of AR genes present are also shown, regardless of detection frequency. Absence of annotated genes does not indicate absence of genes. Genes could have been detected at the empty location but not in greater than 90% of plasmids.

### 3.3. Hierarchal Clustering

**Figure 3:**
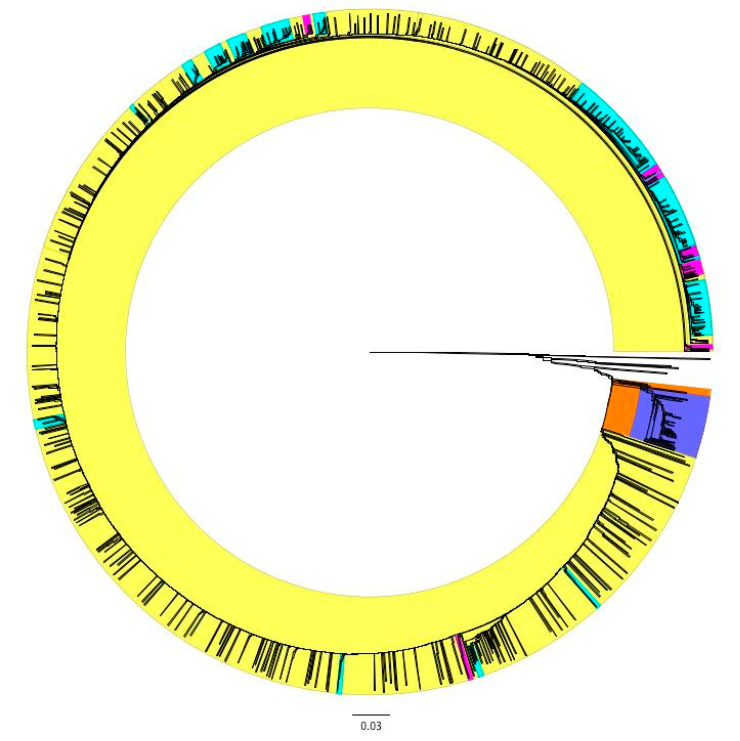
Dendrogram of pESI plasmids (*n* = 2627) based on the presence and absence of genes detected in two or more isolates. Clusters are colored based on the geographic source of the plasmid or the bacterial host. Orange cluster only contained plasmids from Peru and one from Ecuador. Purple cluster is a sub-cluster of the orange cluster containing isolates with a specific group of genes. Yellow cluster contained plasmids from isolates of various sources. Blue clusters contained a majority of plasmids isolated in bacteria collected from U.S. food. Pink cluster contained plasmids from isolates collected from U.S. or international human cases. Scale represents Jaccard distance.

**Figure 4:**
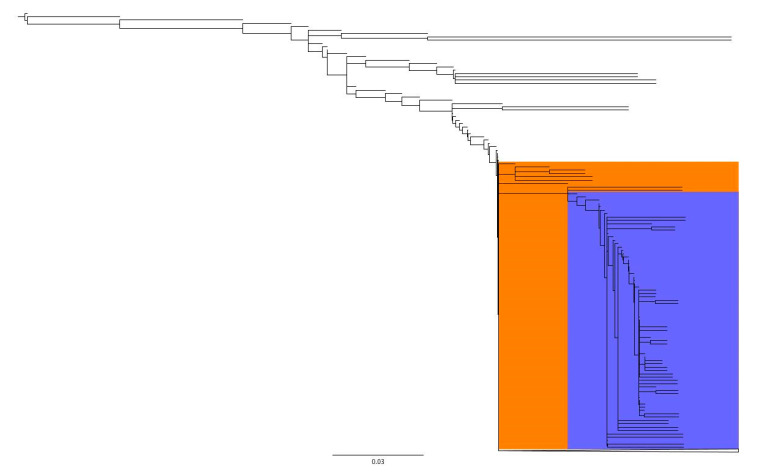
Root of the dendrogram of pESI plasmids based on the presence and absence of genes detected in two or more isolates. Orange cluster only contained plasmids from Peru and one from Ecuador. Purple cluster is a sub-cluster of the orange cluster containing isolates with a specific group of genes. Scale represents Jaccard distance.

Plasmid bins were clustered using the Jaccard Index based on the presence and absence of genes as determined by Roary. Plasmids clustered into several subgroups with only a few groups sharing similar metadata (Figure 3). The majority of plasmids belonged to one large group that divided into many smaller clusters; the few plasmids not included in this large cluster were mostly U.S. food animal isolates collected by USDA-FSIS. Among these plasmids were all of those missing the top four most common genes not present in every plasmid: *parA*, a transcription regulator, a transposase, and hypothetical protein (Figure 3, uncolored portion of dendrogram; Figure 4). The first cluster to split from the large cluster contained isolates from Peru and one from Ecuador (Figure 3, orange and purple clusters; Figure 4). This cluster contained a sub-group comprised of all the plasmids containing the hypothetical backbone target except one isolate that was included in the uncolored cluster (Figure 4, purple cluster). Many of these plasmids were missing genes present in more than 99% of plasmids and contained genes only present in plasmids in this cluster. None of these plasmids contained *bla*_CTX-M-65_.

The remaining plasmids were generally of mixed isolation source and country of origin (Figure 3, yellow cluster). Plasmids did sporadically cluster by isolation source; plasmids from humans clustered with other plasmids from humans and plasmids from animals clustered with other plasmids from animals (Figure 3, pink and blue clusters respectively). The largest of these clusters appeared at the base of the dendrogram.

## 4. Discussion

In order to estimate the carriage rate and gene content variation of the pESI-like plasmid associated with *Salmonella* Infantis, we first analyzed *Salmonella* genomes collected by USDA-FSIS and CDC, as well as publicly available genomes from international sources. While limited to sequenced isolates, the results showed an increase in the carriage rate of the pESI plasmid among *S*. Infantis strains in the U.S. over the time period investigated in isolates from humans, chickens, and cattle and an apparent diversity of pESI-like plasmids based on their gene content.

The carriage rate of the pESI-like plasmid increased in *S*. Infantis isolates from U.S. chickens and cattle from 2017 to 2018. The rate also increased marginally among human infections during the four years analyzed. However, at the sites participating in CDC FoodNet, Infantis infections increased 69% in 2019 from the three previous years and many of those infections were reported to be due to a highly antimicrobial resistant strain, implying that they could contain the pESI plasmid [6].

Despite screening more than 16,000 U.S. *Salmonella* isolates of more than 100 different serotypes, the pESI plasmid was only identified in *S*. Infantis or Infantis variants like Rough 1,5 or I 6,7,:-:,1,5. The pESI plasmid has been suggested to be specific to *S*. Infantis [30]. However, other studies have shown that the plasmid is self-transmissible to *S*. Typhimurium and *E. coli* [17,20]. Transmission was shown to be temperature-dependent, with the highest transfer rate occurring at 37 °C but was possible at 41 °C [17]. The normal body temperature for a chicken is between 39 °C and 41.1 °C depending on age, which potentially allows for transfer of the plasmid between bacterial cells within a chicken host [31]. However, the bile salt concentration of a chicken intestine may inhibit transfer [17]. Rather than serotype specificity, this likely indicates that there is a barrier to conjugation or plasmid maintenance, such as bile salt concentration, entry exclusion systems, or toxin-antitoxin systems, resulting in low to no transfer of the plasmid in an animal host [32,33]. Low or completely inhibited conjugation could explain why no other serotypes have been isolated that contain the pESI plasmid. Genes for conjugation were among the genes present in greater than 95% of plasmids in this analysis, indicating that the vast majority of plasmids should be self-transmissible. Further investigation into the host *S*. Infantis chromosome would be needed to determine whether host-specific factors are promoting plasmid maintenance in serotype Infantis specifically and offering a fitness advantage. Although currently reported only in *S*. Infantis, continued surveillance is also necessary to determine if the pESI plasmid is present or emerges in other *Salmonella* serotypes or bacterial species in the U.S. or other countries.

Only 61% of plasmids in the gene content analysis contained *bla*_CTX-M-65_. Although *bla*_CTX-M-65_ was present on plasmids analyzed as early as 2010 and in almost every country for which isolates were available, the gene was not present in almost 40% of plasmids analyzed. This could mean that if the bacterial host is not under continuous antibiotic pressure the gene is easily lost. Another explanation would be clonal expansion of strains containing plasmids that never carried *bla*_CTX-M-65_. Sequencing efforts often target isolates that are phenotypically resistant, which could have artificially inflated the percentage of international isolates which contained *bla*_CTX-M-65_ investigated in this study. These isolates may not be associated with surveillance programs. This could explain why a higher proportion of isolates from Europe and South America contained the gene than U.S. isolates as the U.S. isolates analyzed were collected through surveillance programs and not part of targeted sequencing efforts.

While *bla*_CTX-M-65_ was a good predictor of pESI plasmid presence in Infantis isolates from U.S. food animals, the absence of *bla*_CTX-M-65_ from an isolate whole genome sequence did not indicate that the isolate did not carry the pESI plasmid. However, used in combination, the other target sequences were better indicators that the pESI plasmid was absent from an isolate. All plasmids in the carriage rate analysis contained the I1 relaxase target, the *trbA* target, and the Ybt target in addition to the pESI *repA* target which was required for judgement that the isolate contained at least a partial pESI plasmid. The K88 and *ipf* targets were also present in greater than 99% of plasmids in the gene variation analysis. The *pilL*, *ardA*, and *sogS* targets were present in greater than 95% of isolates and the *merA* target was present in greater than 90% in the gene variation analysis. The hypothetical backbone target, which was only present in two isolates collected by CDC in 2016 in the carriage rate analysis, was present in less than 3% of isolates, all isolated in Peru prior to 2013. In the future, the hypothetical backbone protein target should not be used to indicate pESI plasmid presence or absence.

In the gene content analysis the IncP target was divided into two separate gene groups, present in less than 35% of isolates in both cases, and did not include the entire sequence. The IncP target also split into two hits in the carriage rate analysis. This likely indicates that the assembly program split contigs at the IncP target, so while the nucleotide-based carriage rate analysis was able to identify the entire sequence, the annotation-based analysis was not. The K88 target sequence was also only identified as a partial gene in the variation analysis as well, indicating a similar problem. This may indicate that other genes were split into two genes elsewhere in the Roary analysis, allowing for the possibility of Roary artificially inflating the number of genes present.

Several genes present in greater than 90% of plasmids could explain the success of *S*. Infantis containing the pESI plasmid in a poultry host. The first report of the pESI plasmid in 2014 showed that the pESI plasmid contained several fimbriae genes (including two novel genes) that were associated with higher rates of adhesion in human and chicken cells and increased virulence in mouse models. This study also reported presence of the yersiniabactin siderophore operon (Ybt) which was inducible under low iron conditions [18]. Iron is a growth-limiting factor for many bacteria and acquisition can play a role in pathogenesis for some pathogens, including some associated with poultry, like avian pathogenic *E. coli*, *Clostridium perfringens*, and *Campylobacter jejuni* [34,35,36]. Low iron levels have also been shown to restrict the growth of *Salmonella* in both eggs and live poultry, thus an iron acquisition system like Ybt would give *Salmonella* strains an advantage over strains that lacked them [37,38]. Greater than 99% of plasmids in the gene content analysis contained the entire Ybt operon and several fimbriae genes. The remaining fimbriae genes were detected in greater than 90% of plasmids, with the majority detected in greater than 95%. Increased ability to acquire iron would give this strain a survival advantage in a poultry host.

In addition to virulence factors identified in the first report of the pESI plasmid, between 77 and 83% of plasmids contained genes for resistance to arsenic, but it is unknown if any of the isolates are phenotypically resistant to arsenic. Feed containing organic arsenic was voluntarily withdrawn in the U.S. completely as of 2016 but may still be used internationally [39]. However, *S*. Infantis containing the pESI plasmid was detected in U.S. poultry isolates as early as 2014; if functional, these genes could have conferred an advantage prior to the withdrawal. More than 92% of plasmids also contained at least one antibiotic resistance gene. The use of antibiotics in animal feed and water was discontinued in the U.S. prior to the emergence of the pESI plasmid [40]. While antibiotics are approved for the treatment of sick animals, 51% of the chickens raised in the U.S. in 2018 were raised with no antibiotic exposure [41]. Further, use of cephalosporins, including those *bla*_CTX-M-65_ confer resistance to, is prohibited in food animals in the U.S. [42]. Further investigation would be needed to assess the impact of the resistance genes for arsenic and antibiotics as fitness advantages.

In the hierarchical clustering analysis, the majority of plasmids did not cluster based on animal source or geographic source. However, one cluster of plasmids, all from South America isolated prior to 2013, were the only plasmids in the gene content analysis that contained the hypothetical backbone target but did not contain *bla*_CTX-M-65_. This cluster of plasmids contained a group of genes, including an entry exclusion protein and hypothetical proteins, that no other plasmids contained but lacked genes that were detected in greater than 95% of the other plasmids, including a different entry exclusion protein. This could indicate a separate lineage of plasmids: one lineage circulating in South America and Italy that contains this hypothetical gene but not *bla*_CTX-M-65_, and the lineage that has been introduced to the U.S. poultry population that does contain *bla*_CTX-M-65_, but not the hypothetical backbone target. A recent study of European Infantis isolates concluded that the pESI plasmid containing *bla*_CTX-M-65_ was unrelated to the plasmids circulating in European isolates, although only a few plasmids from the U.S were included in the study [43].

The international isolates investigated in this study provide a good baseline for studying this plasmid, but more isolates are needed. The pESI and pESI-like plasmids have been reported in Israel, Italy, Peru, Turkey, Switzerland, Hungary, Russia, and the U.S. [12,14,16,18,19,20,30,44,45]. Infantis isolates collected in Japan were predicted to contain the pESI plasmid based on the presence of the *irp*2 gene [46]. Cases of *S*. Infantis carrying *bla*_CTX-M-65_ on a plasmid were reported in Ecuador in 2014–2015 [13]. *S*. Infantis carrying *bla*_CTX-M-65_ was isolated from a wild owl in Chile [15]. Isolates collected from Peru, Colombia, Ecuador, Venezuela, Switzerland, the United Kingdom, Vietnam, and Chile were investigated in this study. However, the plasmid could be circulating in countries for which isolate sequences were not available; hence, examining isolates from additional countries will be critical to understanding the global spread of this plasmid.

## 5. Conclusions

The carriage rate of the pESI-like plasmid increased in *S*. Infantis isolates collected from U.S. chickens and human clinical cases from 2017 to 2018. Plasmids isolated in *S*. Infantis in the U.S. are similar to plasmids that have been sequenced from other countries. Surprisingly, the *bla*_CTX-M-65_ gene was only detected in about 60% of isolates with the prevalence varying based on the country of origin and host species. This demonstrates the importance of surveillance for different genes associated with the plasmid and not just *bla*_CTX-M-65_, as other genes on the plasmid could confer an advantage in a poultry or human host. Additional studies will be needed to understand the advantages the pESI-like or similar plasmids may confer to the host bacteria. Further insight into the issues and findings highlighted in this study can potentially help in developing specific on-farm mitigation strategies for the zoonotic pathogens that may harbor pESI or other plasmids.

## Figures and Tables

**Table 1:** Target genes used to determine pESI presence in the carriage analysis.

Target	Target Function
ardA *	Restriction modification enzyme
*traI*	Relaxase (IncI1)
sogS *	Primase (IncI1)
trbA *	Conjugal transfer protein (IncI1)
pESI *repA*	Plasmid replication
*bla*_CTX-M-65_	Extended Spectrum Betalactamase
IncP	Iterons associated with IncP
hypothetical backbone protein *	Hypothetical protein associated with pESI
K88 *	Fimbriae
Ybt *	*irp*2 (Ybt operon); yersiniabactin biosynthesis
*merA* *	Mercuric reductase (*mer* operon)
*ipf* *	Fimbriae
*pilL* *	Pilus biosynthesis (IncI1)

* From reference [12].

**Table 2:** pESI target profiles of isolates collect by United States Department of Agriculture-Food Safety and Inspection Service Hazard Analysis and Critical Control Point sampling. Plus indicates present, minus indicates absent.

Targets Present in Isolates Containing the pESI Plasmid												2017			2018			
												Chicken		Turkey	Chicken		Cattle	Turkey
I1	pESI *repA*	*ipf*	IncP	*bla*_CTX-M-65_	*ardA*	*pilL*	*sogS*	*trbA*	K88	*merA*	Ytb	Infantis	Rough 1,5-	Infantis	Infantis	Rough 1,5-	Infantis	Infantis
+	+	+	+	+	+	+	+	+	+	+	+	111	4	4	103	3	1	5
+	+	+	+	+	+	+	+	+	+	-	+	1	0	0	1	0	0	0
+	+	+	+	+	+	+	+	+	-	+	+	14	1	1	33	1	1	4
+	+	+	+	-	+	+	+	+	+	+	+	102	4	4	130	6	0	5
+	+	+	+	-	+	+	+	+	-	+	+	11	1	0	34	2	0	0
+	+	+	+	-	+	+	+	+	-	-	+	1	0	0	0	0	0	0
+	+	+	+	-	-	+	+	+	+	+	+	1	0	0	0	0	0	0
+	+	+	+	-	-	+	+	+	-	+	+	1	0	0	1	0	0	0
+	+	+	-	+	+	+	+	+	+	-	+	5	0	0	5	0	0	0
+	+	+	-	+	+	+	+	+	-	+	+	0	0	0	1	0	0	0
+	+	+	-	+	+	+	+	+	-	-	+	2	0	0	0	0	0	0
+	+	+	-	+	+	-	+	+	+	-	+	5	0	0	2	0	0	0
+	+	+	-	+	+	-	+	+	-	-	+	0	0	0	1	0	0	0
+	+	+	-	+	+	-	-	+	+	-	+	0	0	0	2	0	0	0
+	+	+	-	+	+	-	-	+	-	-	+	0	0	0	2	0	0	0
+	+	+	-	-	+	+	+	+	+	+	+	1	0	0	0	0	0	0
+	+	+	-	-	+	+	+	+	+	-	+	2	0	0	10	0	0	0
+	+	+	-	-	+	+	+	+	-	-	+	2	0	0	2	0	0	0
+	+	+	-	-	+	-	+	+	+	-	+	2	0	0	5	0	0	1
+	+	+	-	-	+	-	+	+	-	-	+	0	0	0	1	0	0	0
+	+	-	+	+	+	+	+	+	+	+	+	0	0	0	4	0	0	0
+	+	-	+	+	+	+	+	+	-	+	+	0	0	0	1	0	0	0
+	+	-	+	+	+	-	+	+	-	+	+	0	0	0	1	0	0	0
+	+	-	+	-	+	+	+	+	+	+	+	0	0	0	2	0	0	0
+	+	-	+	-	+	+	+	+	-	+	+	0	0	0	3	0	0	0
+	+	-	-	+	+	+	+	+	+	-	+	0	0	0	1	0	0	0
Total												261	10	9	345	12	2	15

**Table 3:** pESI target profiles of the Centers for Disease Control and Prevention National Antimicrobial Resistance Monitoring System isolates. Plus indicates present, minus indicates absent.

Targets Present in Isolates Containing the pESI Plasmid													2015	2016	2017	2018		
I1	pESI *repA*	*ipf*	IncP	*bla*_CTX-M-65_	*ardA*	*pilL*	*sogS*	*trbA*	K88	*merA*	Ytb	hyp	Infantis		Infantis *	Infantis	I 6,7:-:,1,5	Rough 1,5
+	+	+	+	+	+	+	+	+	+	+	+	+	0	3	0	0	0	0
+	+	+	+	+	+	+	+	+	+	+	+	-	0	0	15	14	2	1
+	+	+	+	+	-	+	+	+	+	+	+	-	0	0	0	1	0	0
+	+	+	+	-	+	+	+	+	+	+	+	-	0	1	5	7	2	0
+	+	+	-	+	+	-	+	+	+	-	+	-	0	0	1	1	0	0
+	+	+	-	+	+	+	+	+	+	-	+	-	0	0	0	1	0	0
+	+	+	-	-	+	+	+	+	+	-	+	-	0	0	0	1	0	0
Total													0	4	21	25	4	1

* One isolate was initially called serotype Nigeria.

## Supplementary Materials

The following are available online at https://www.mdpi.com/2073-4425/11/12/1516/s1, Table S1: Target sequences used to determine pESI presence in isolates collected for NARMS, Table S2: Details of isolates included in the pan-plasmid genome analysis, Table S3: Genes present in greater than 99% of isolates with locations relative to the genes in CP016407.1. Hypothetical proteins are not listed. tnp indicates unnamed transposase gene. Gene names were identified using BLAST, Table S4: Genes present in greater than 95% and less than 99% of isolates with locations relative the genes in CP016407.1. Hypothetical proteins are not listed. tnp indicates unnamed transposase gene. Gene names were identified using BLAST, Table S5: Genes present in greater than 90% and less than 95% of isolates with locations relative the genes in CP016407.1. Hypothetical proteins are not listed. tnp indicates unnamed transposase gene. Antibiotic resistance genes present in less than 90% of isolates are also listed with the percentage of isolates that contain them in brackets next to the gene name. Gene names were identified using BLAST.

## Note

The mention of trade names or commercial products in this manuscript is solely for the purpose of providing specific information and does not imply recommendation or endorsement by the U.S. Department of Agriculture.
